# Effect of exercise intensity on metabolic responses on combined application of electrical stimulation and voluntary exercise

**DOI:** 10.14814/phy2.14758

**Published:** 2021-02-15

**Authors:** Kohei Watanabe, Tatsuya Takada, Shuhei Kawade, Toshio Moritani

**Affiliations:** ^1^ Laboratory of Neuromuscular Biomechanics Faculty of Liberal Arts and Sciences and School of International Liberal Studies Chukyo University Nagoya Japan; ^2^ MTG Co., Ltd. Nagoya Japan; ^3^ School of Health and Sport Sciences Chukyo University Toyota Japan; ^4^ Faculty of Sociology Kyoto Sangyo University Kyoto Japan

**Keywords:** blood lactate concentration, electrical muscle stimulation, electrical myostimulation, energy metabolism

## Abstract

The combined application of voluntary exercises and neuromuscular electrical stimulation (NMES) has been developed as a new type of exercise that can recruit motor units contributing to both aerobic and anaerobic energy metabolisms. We aimed to investigate the effect of voluntary exercise intensity on metabolic responses on the combination of voluntary exercise and NMES. In 13 volunteers, oxygen consumption and the blood lactate concentration were measured during (1) voluntary pedaling exercise at four different intensities: 50%, 75%, 100%, and 125% of the ventilatory threshold (VT) (VOL), (2) these voluntary exercises with superimposed NMES applied to the gluteus and thigh muscles (VOL+NMES), and (3) NMES only (NMES). Oxygen consumption and the blood lactate concentration in VOL+NMES were significantly greater than VOL at each exercise intensity (*p* < 0.05). Differences in oxygen consumption between VOL+NMES and VOL decreased with exercise intensity, and that at 125% VT was significantly lower than the net gain in oxygen consumption following NMES (*p* < 0.05). Differences in the blood lactate concentration between VOL+NMES and VOL increased with exercise intensity, and that at 50% VT was significantly lower than the net gain in the blood lactate concentration following NMES (*p* < 0.05). Our results suggest that voluntary exercise intensity has a critical impact on metabolic responses during the combined application of voluntary exercises and NMES. Superimposing NMES onto voluntary exercises at high exercise intensities may induce overlapping recruitment of motor units, leading to a markedly reduced benefit of additional metabolic responses on its superimposition.

## Graphical abstract

We aimed to investigate the effect of voluntary exercise intensity on metabolic responses on the combination of voluntary exercise and NMES. Superimposing NMES onto voluntary exercises at high exercise intensities may induce overlapping recruitment of motor units, leading to a markedly reduced benefit of additional metabolic responses on its superimposition.

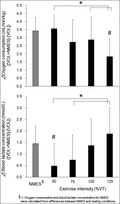

## 1 INTRODUCTION

The combined application of voluntary aerobic exercises and neuromuscular electrical stimulation (NMES) has recently been developed and recognized as a new form of exercise (Paillard, 2008; Paillard, 2018; Kemmler et al., 2010; Kemmler et al., 2012). Motor units are orderly recruited from low‐threshold motor units during voluntary exercises, while NMES induces preferential recruitment of high‐threshold motor units or random‐order recruitment of low‐ and high‐threshold motor units (Bickel et al., 2011; Gregory & Bickel, 2005; Jubeau et al., 2007). Thus, the combination of voluntary exercise and NMES should activate the motor units which are recruited during both low‐ and high‐intensity exercises, and thereby could induce metabolic responses of low‐ and high‐intensity exercises simultaneously. This hybrid method may be useful for older adults and patients with metabolic diseases, that is, type 2 diabetes mellitus, who require high‐intensity exercises such as resistance exercises for muscle hypertrophy and the control of blood glucose and insulin resistance, in addition to low‐intensity aerobic exercises (Kemmler et al., 2014, 2018). The combined application of voluntary aerobic exercises and NMES could also be convenient for people who do not have opportunities to perform both aerobic and resistance/anaerobic exercises due to time constraints.

Previous studies, including our work, already reported that the combined application of NMES and voluntary aerobic exercises can increase oxygen consumption and enhance glucose metabolism because of the additional NMES‐elicited recruitment of motor units that are not activated for given voluntary exercises and/or high‐threshold motor units (Kemmler et al., 2012; Watanabe et al., 2014, 2019). On the other hand, the benefit of superimposing NMES onto voluntary exercises would be reduced with high‐intensity, anaerobic, or resistance exercises in terms of the additional recruitment of motor units and its induced metabolic responses, since any additional recruitment of motor units by superimposing NMES is theoretically impossible when all motor units are fully recruited, as in the case of maximal voluntary contraction (Hortobagyi et al., 1992; Koutedakis et al., 1995). Therefore, it is reasonable to consider that physiological responses and the benefits of combining voluntary aerobic exercises with NMES would be influenced by the intensity of the voluntary exercises.

The aim of the present study was to investigate the effect of voluntary exercise intensity on metabolic responses during the combined application of voluntary pedaling exercise and NMES. We hypothesized that increases/changes in metabolic responses following the superimposing of NMES would be attenuated during high‐intensity voluntary exercise exceeding the anaerobic threshold because of the overlapping recruitment of motor units/muscle fibers with high‐recruitment thresholds between voluntary exercise and NMES.

## 2 Methods

### 2.1 Participants

Thirteen healthy young adults (age: 21.2 ± 1.0 years, height: 168.0 ± 5.7 cm, body mass: 57.4 ± 5.7 kg, 10 men and 3 women) volunteered for the present study. They did not participate in regular endurance/strength training or competitive athletic events. All subjects gave written informed consent for the study after receiving a detailed explanation of the purposes, potential benefits, and risks associated with participation. They were healthy with no history of any metabolic, musculoskeletal, or neurological disorders. All study procedures were conducted in accordance with the Declaration of Helsinki and research code of ethics of Chukyo University, and were approved by the Committee for Human Experimentation of Chukyo University (2017–002 and −057).

### 2.2 Design

Subjects visited the laboratory four times, separated by at least 48‐h intervals. On the first day, an exercise tolerance test using a cycle ergometer (75XL II; Combi) was performed to determine the exercise intensity at the ventilatory threshold (VT) and peak oxygen consumption for each participant. The workload in the exercise tolerance test was increased by 15 W every 1 min starting from 60 W for men and 40 W for women. The participants were instructed to maintain the pedaling exercise at a 60‐rpm cadence until reaching a point meeting one of two criteria: (a) oxygen consumption (VO_2_) reached a steady‐state despite a load increase or (b) subjects could not maintain pedaling at 60 rpm, to determine peak oxygen consumption. VT was estimated using respiratory gas exchange parameters, for example, the point of departure from linearity in VE and VO_2_, an abrupt increase of the expiratory O_2_ fraction, a systematic increase in the ventilatory equivalent for O_2_ (VE/VO_2_) without any increase in the ventilatory equivalent for CO_2_ (VE/VCO_2_), and a systematic increase in the end‐tidal O_2_ partial pressure (PETO_2_) without any decrease in the end‐tidal CO_2_ partial pressure (PETCO_2_) (Raven et al., 2014; Wasserman, 1984).

**FIGURE 1:**
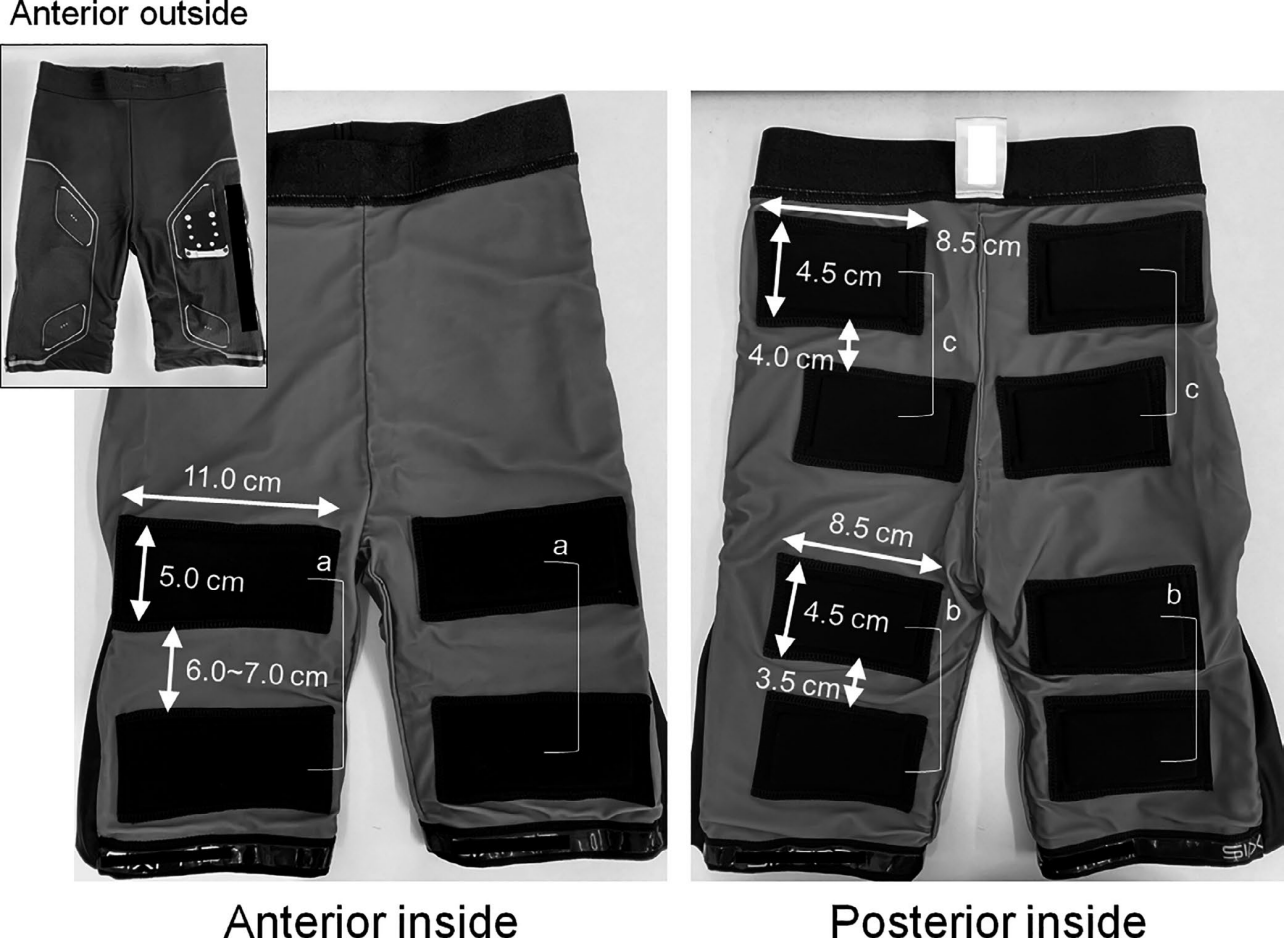
Electrodes for neuromuscular electrical stimulation attached to the inside of shorts. (A), (B), and (C) indicate electrode pairs for quadriceps femoris, hamstrings, and gluteus muscles, respectively.

On the second day, NMES was administered in a supine position on a bed without performing any voluntary contractions for 3 min (NMES). NMES was applied to anterior and posterior thigh muscles and gluteus muscles using a custom‐made stimulator based on a commercially developed NMES device (SIXPAD, MTG Ltd., Nagoya, Japan) (Watanabe et al., 2017, 2019) and silicon‐rubber electrodes covered by wet patches of material fixed on the inside of fitted shorts (Figure 1) (Watanabe et al., 2019). NMES for all electrode pairs was synchronized and biphasic square current pulses with a 100‐μs duration were then constantly applied with a 4‐Hz stimulation frequency. The NMES intensity was set at the maximum intensity at which participants could perform the pedaling movements without discomfort or stress (50 V and 4.85 mA).

On the third and fourth days, the participants performed pedaling exercise on a cycle ergometer at four different workloads: 50%, 75%, 100%, and 125% of VT. Each exercise was performed for 3 min at a 60‐rpm cadence. The order of the four different workloads on the third or fourth day was randomized and >20‐min rest intervals were set between the exercises. The participants performed pedaling exercise as voluntary exercise (VOL) without superimposed NMES on the third day and NMES was superimposed onto these pedaling exercises (VOL+NMES) on the fourth day. Parameters of NMES were the same as those on the second day and were not changed across the different intensities in VOL+NMES.

### 2.3 Measurements

During all exercises on the first to fourth days, expired gas was measured from a mask covering the mouth and nose using the breath‐by‐breath method (AE310S, Minato Medical Science Co., Ltd.) (Watanabe et al., 2014, 2019). VO_2_ relative to the body mass and the respiratory gas exchange ratio (RER) were calculated from the expired gas sampled for the final 1 min of 3‐min exercises. The blood lactate concentration was measured from two 5‐μL samples of blood obtained from the fingertip just before and after the exercises with the lactate oxidase method using an automated analyzer (Lactate Pro; Arkray) (Watanabe et al., 2014, 2019). We also measured the rate of perceived exertion (RPE) on a Borg scale just before the end of each exercise for VOL and VOL+NMES.

### 2.4 Data and statistical analyses

All data are presented as the mean and standard deviation. Before the statistical analysis, the normality of the data distribution was tested by the Shapiro–Wilk test. When a normal distribution was confirmed, parametric tests were used for further analysis. Nonparametric analysis was used when a normal distribution was not confirmed. To test the effects of additional NMES and exercise intensity and their interaction, two‐way repeated‐measures ANOVA (with/without NMES vs. exercise intensity) was applied to oxygen consumption and the blood lactate concentration for VOL and VOL+NMES. Oxygen consumption and the blood lactate concentration were also compared between VOL and VOL+NMES at each exercise intensity by the paired *t*‐test. To test the effect of the exercise intensity on RER for VOL and VOL+NMES, Friedman's test was used since the data distribution was not normal. RER and RPE at each exercise intensity were compared between VOL and VOL+NMES by the Wilcoxon signed‐rank test. To quantify the effect of superimposing NMES onto voluntary exercise, differences between VOL and VOL+NMES in oxygen consumption and the blood lactate concentration were calculated and compared with differences between NMES and the resting condition (net gains following NMES) with the same parameters using the paired *t*‐test. Also, differences between VOL and VOL+NMES in oxygen consumption and the blood lactate concentration were compared among the exercise intensities by Tukey's HSD test following one‐way repeated‐measures ANOVA. In order to confirm the physiological conditions before the session, blood lactate concentrations before the session were also compared among the different exercise intensities within VOL or VOL+NMES by Friedman test and between VOL or VOL+NMES at each intensity by Wilcoxon signed‐rank test.

To test the effect size, the eta‐squared statistic (η^2^), the Kendall's W test (W), Cohen's index (d), and correlation coefficient were analyzed for one‐ and two‐way repeated‐measures ANOVA, Friedman test, paired *t*‐test, and Wilcoxon signed‐rank test (Tomczak & Tomczak, 2014). We also calculated statistical power for post hoc tests after ANOVA tests and Friedman test (Vincent, 2005).

All statistical analyses were performed using SPSS software (SPSS version 11.5; SPSS).

## 3 RESULTS

The mean peak oxygen consumption (VO_2_ peak) was 44.8 ± 6.0 mL/min/kg. The mean percentage of peak oxygen consumption and workload at VT were 63.4 ± 5.2% and 124.6 ± 19.2 W, respectively. The exercise intensities at 50, 75, 100, and 125% of VT were 62.3 ± 9.6, 93.5 ± 14.4, 124.6 ± 19.2, and 155.8 ± 24.0 W, respectively.

**FIGURE 2:**
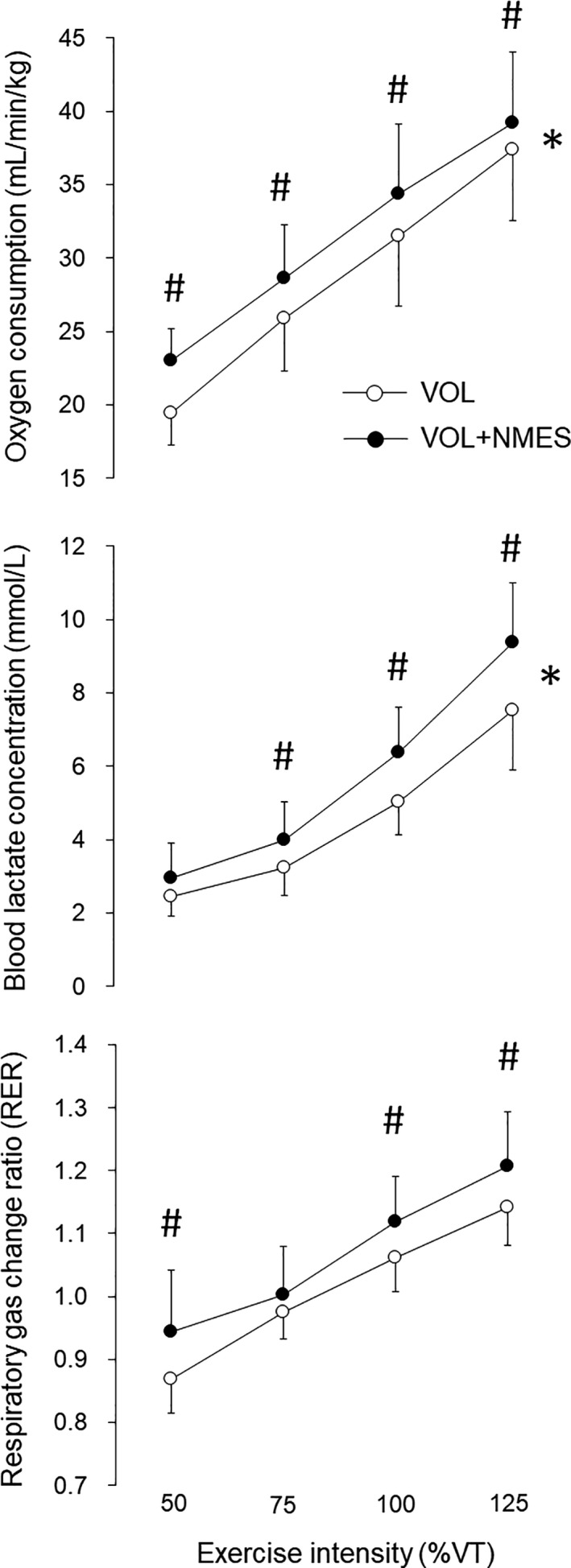
Oxygen consumption, blood lactate concentration, and respiratory gas change ratio (RER) for voluntary pedaling exercise (VOL) and the combination of voluntary pedaling exercise and neuromuscular electrical stimulation (VOL+NMES) at various exercise intensities. * *p* < 0.05 interaction with/without NMES vs. exercise intensity, # *p* < 0.05 between VOL and VOL+NMES.

Oxygen consumption, the blood lactate concentration, and RER for VOL and VOL+NMES are shown in Figure 2. There were significant interactions (with/without NMES vs. exercise intensity) and significant effects of NMES and exercise intensity on oxygen consumption (*p* < 0.05, η^2^ = 0.07) and the blood lactate concentration for VOL and VOL+NMES (*p* < 0.05, η^2^ = 0.058) (upper and middle panels in Figure 2). Significant differences between VOL and VOL+NMES were observed in oxygen consumption at all exercise intensities (*p* < 0.05, d = 1.28, 0.75, 0.62, and 0.39 for 50%, 75%, 100%, and 125%) (upper panel in Figure 2) and the blood lactate concentration at 75%, 100%, and 125% of VT (*p* < 0.05, d = 0.35, 0.95, and 0.74 for 75%, 100%, and 125%) (middle panel in Figure 2). For RER, significant effects of exercise intensity were noted for both VOL and VOL+NMES (*p* < 0.05, W = 8.47 and 8.15 for VOL and VOL+NMES), and significant differences between VOL and VOL+NMES were detected at 50%, 100%, and 125% of VT (*p* < 0.05, r = 0.58, 0.85, and 0.84 for 50%, 100%, and 125%) (lower panel in Figure 2).

Oxygen consumption for VOL and VOL+NMES corresponded to 43.8 ± 4.6 and 51.9 ± 5.6% of the VO_2_ peak at 50% of VT, 58.2 ± 6.9 and 64.4 ± 6.6% of the VO_2_ peak at 75% of VT, 70.6 ± 8.2 and 77.1 ± 6.8% of the VO_2_ peak at 100% of VT, and 83.8 ± 7.5 and 88.0 ± 7.8% of the VO_2_ peak at 125% of VT, respectively. There were no significant differences in RPE between VOL and VOL+NMES at each exercise intensity (*p* > 0.05).

For NMES, oxygen consumption, the blood lactate concentration, and RER were 6.9 ± 0.8 mL/min/kg (15.6 ± 1.5% of VO_2_ peak), 2.7 ± 0.7 mmol/L, and 0.92 ± 0.07, respectively.

**FIGURE 3:**
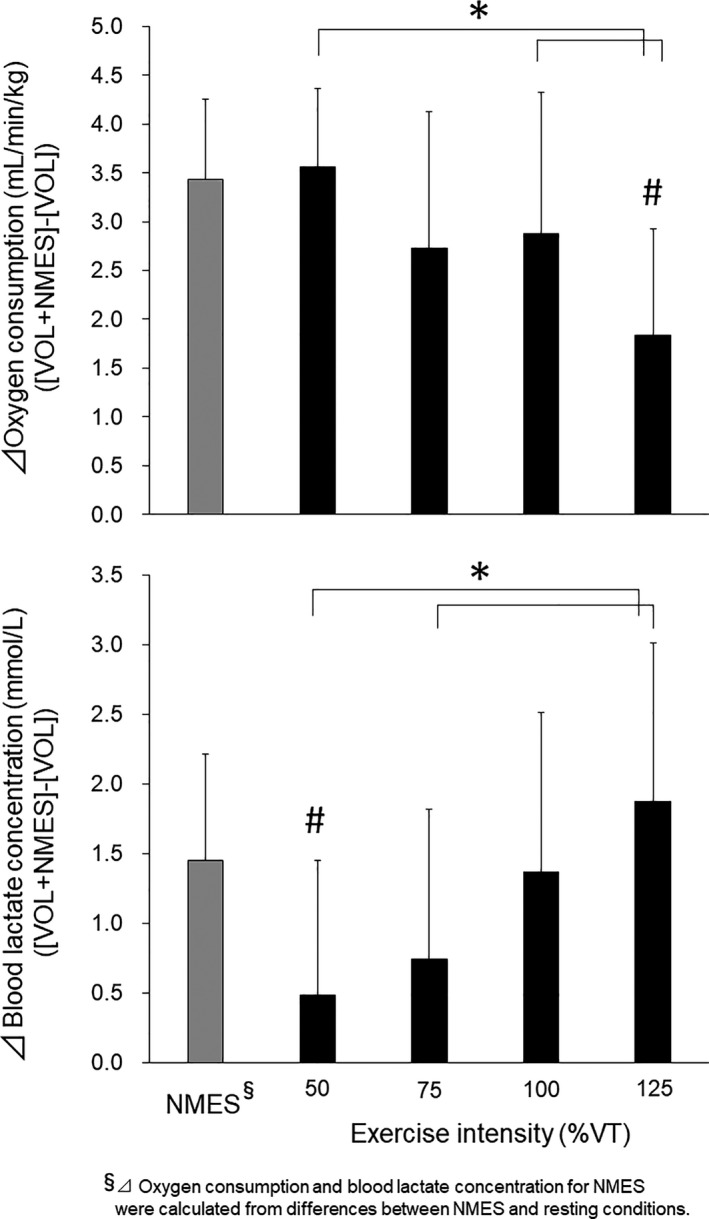
Differences in oxygen consumption (upper panel) and the blood lactate concentration (lower panel) between neuromuscular electrical stimulation (NMES) and the resting condition (left gray bars) and between voluntary pedaling exercise (VOL) and the combined application of voluntary pedaling exercise and neuromuscular electrical stimulation (VOL+NMES) (Black bars). * *p* < 0.05 between the intensities, # *p* < 0.05 vs. NMES.

Significant effects of exercise intensity were observed regarding differences in oxygen consumption and the blood lactate concentration between VOL and VOL+NMES (*p* < 0.05, η^2^ = 0.236 and 0.207 for oxygen consumption and the blood lactate concentration) (Figure 3). The difference in oxygen consumption between VOL and VOL+NMES at 125% of VT was significantly lower than those at 50% and 100% of VT (*p* < 0.05, d = 0.22 and 0.26 for 50% and 100%) (upper panel in Figure 3). Significantly greater differences in the blood lactate concentration between VOL and VOL+NMES at 125% of VT were noted when compared with those at 50 and 75% of VT (*p* < 0.05, d = 0.14 and 0.06 for 50 and 75%) (Lower panel in Figure 3). The difference in oxygen consumption between VOL and VOL+NMES at 125% of VT was significantly lower than that in oxygen consumption between NMES and the resting condition (*p* < 0.05, d = 1.27) (Upper panel in Figure 3). The difference in the blood lactate concentration between VOL and VOL+NMES at 50% of VT was significantly lower than that in the blood lactate concentration between NMES and the resting condition (*p* < 0.05, d = 0.99) (Lower panel in Figure 3).

There were no significant effects of exercise intensity and no significant differences between VOL and VOL+NMES in blood lactate concentration before the sessions (*p* > 0.05) (VOL: 1.23 ± 0.23, 1.28 ± 0.23, 1.22 ± 0.25, and 1.08 ± 0.27 at 50%, 75%, 100%, and 125% of VT, VOL+NMES: 1.39 ± 0.26, 1.38 ± 0.37, 1.29 ± 0.35, and 1.30 ± 0.35 at 50%, 75%, 100%, and 125% of VT).

Statistical power for oxygen consumption, the blood lactate concentration, RER, and RPE in comparison between VOL and VOL+NMES were 78.6 ± 15.6, 70.1 ± 8.4, 60.6 ± 16.0, and 86.8 ± 11.7. For comparisons in differences between VOL and VOL+NMES at 50%, 75%, 100%, and 125% of VT and its differences between NMES and the resting condition, statistical power for oxygen consumption, the blood lactate concentration were 84.8 ± 8.2 and 78.0 ± 15.9.

## 4 DISCUSSION

Increases in oxygen consumption following the superimposing of NMES onto voluntary exercise were noted in the present study regardless of the voluntary exercise intensity (upper panel in Figure 2). This result is in agreement with previous studies (Kemmler et al., 2012; Watanabe et al., 2014, 2019) and could be explained by the additional recruitments of motor units and muscle fibers that were not activated during the given voluntary exercises. Our study also showed that increments in oxygen consumption following the superimposed NMES were influenced by the voluntary exercise intensity (significant interaction in with/without NMES vs. exercise intensity) (upper panel in Figure 2), and the increment in oxygen consumption at 125% of VT was significantly lower than those at lower exercise intensities (upper panel in Figure 3). These results support our hypothesis that increases in metabolic responses following the superimposing of NMES would be attenuated during high‐intensity voluntary exercises. Moreover, we observed significantly lower differences in oxygen consumption between VOL and VOL+NMES at 125% of VT compared with the net oxygen consumption of NMES, estimated from the difference in oxygen consumption between NMES and the resting condition (*p* < 0.05) (upper panel in Figure 3). This means that the gain in metabolic responses following the superimposing of NMES was lost when the voluntary exercise intensity exceeded the anaerobic threshold, and this may reflect a failure of NMES‐elicited muscle contractions due to overlapping recruitment of motor units between voluntary exercise and NMES. Previous studies suggested that superimposed NMES cannot induce additional muscle contractions during maximal voluntary contraction, which fully recruits all motor units/muscle fibers (Hortobagyi et al., 1992; Koutedakis et al., 1995). Also, from the results of glycogen depletion measured in human vastus lateralis muscles during pedaling exercises, it could be estimated that slow‐twitch muscle fibers are activated throughout low to high exercise intensities, while fast‐twitch muscle fibers are recruited from 50% of VO_2_ max, and all muscle fibers are fully recruited at over 75% of VO_2_ max (Vollestad & Blom, 1985). Since oxygen consumptions at 50%, 75%, 100%, and 125% of VT corresponded to 44%, 58%, 71%, and 84% of VO_2_ peak for VOL in the present study, respectively, we considered that most muscle fibers would be recruited from 75% of VT and be fully activated at 125% of VT in the present study. We thus concluded that the superimposed NMES during voluntary exercise over the anaerobic threshold, that is, VT, would not elicit additional motor unit recruitment/muscle fiber contractions or associated enhancements of metabolic responses.

The blood lactate concentration and RER during NMES in the present study closely mimicked responses to voluntary exercises at middle to high exercise intensities, although oxygen consumption during NMES was very low (6.9 ± 0.8 mL/min/kg gross; approximately 3.4 mL/min/kg net). Similar results were observed in previous studies (Hamada et al., ,^2003^; Hamada, Hayashi, et al., 2004; Kim et al., 1995; Vanderthommen et al., 2003). We thus considered that limited numbers of motor units with high recruitment thresholds and fast‐twitch/glycolytic type II muscle fibers were recruited following NMES in this study. This would also be supported by nonsignificant differences in the increments of oxygen consumption following NMES in VOL+NMES at 50%–100% of VT when compared with the net gain in oxygen consumption following NMES (upper panel in Figure 3). In the case that motor units with low‐mid recruitment thresholds are mainly recruited following NMES like a voluntary contraction following the size‐principle (Henneman et al., 1965), differences in oxygen consumption between VOL+NMES and VOL at low voluntary exercise intensities should be smaller than the net gain of NMES because of the overlapping recruitment of motor units with lower recruitment thresholds. Theoretically, motor units with high recruitment thresholds and fast‐twitch muscle fibers are preferentially activated during NMES because their larger nerve axons have a much lower electrical resistance for a given externally applied electric current (Clamann et al., 1974). This electrophysiological theory was supported by enhancements of glucose and anaerobic metabolism during NMES compared with voluntary exercises in humans (Hamada, Hayashi, et al., 2004; Hamada et al., 2003; Kim et al., 1995; Vanderthommen et al., (1985). 2003) and by an experiment involving an intramuscular electromyography technique in human skeletal muscle (Hamada, Kimura, et al., 2004). On the other hand, it has been pointed out that this “reversal of the size‐related orderly motor unit recruitment” is only imposed by nerve stimulation and NMES applied via the skin surface inducing random and spatially fixed recruitment of motor units (Gregory & Bickel, 2005; Jubeau et al., 2007; Maffiuletti et al., 2011). These opinions are supported by glycogen depletion in all muscle fiber types during NMES (Johnson et al., 2003) and muscle hypertrophy in both type I and II fibers following NMES intervention (Natsume et al., 2018). Also, interindividual variations in the order of motor unit recruitment during NMES were reported in humans on measuring the conduction velocity of motor unit action potentials: a similar recruitment order between NMES and voluntary contraction was shown in 23 cases and a reverse order was noted in 9 cases (Knaflitz et al., 1990). From these previous studies, we suggest that the motor unit recruitment pattern during NMES is markedly different from that during voluntary exercises and may vary depending on NMES procedures or materials and participants' characteristics. Although it is difficult to identify the detailed motor unit recruitment patterns during the exercises, we concluded that motor units with high recruitment thresholds were recruited following the superimposing of NMES in the present study based on the results of oxygen consumption, the blood lactate concentration, and RER.

The blood lactate concentration and RER were significantly increased by superimposing NMES onto the voluntary pedaling exercise at various exercise intensities (middle and lower panels in Figure 2). These findings support the results of previous studies (Watanabe et al., 2014, 2019) and could reflect the recruitment of motor unit/muscle fibers contributing to glucose metabolism. However, at 50% of VT, there was no significant difference in the blood lactate concentration between VOL and VOL+NMES (middle panel in Figure 2), and the increase in the blood lactate concentration during VOL+NMES was significantly lower than the net gain in the blood lactate concentration induced by NMES (lower panel in Figure 3). Since RER for VOL+NMES was significantly greater than that for VOL at 50% of VT (lower panel in Figure 2), glucose/anaerobic metabolism would be enhanced by superimposing NMES. Both the production and consumption of lactate in metabolic systems are reflected in the blood lactate concentration, and lactate can be utilized as an energy source by skeletal muscles for aerobic exercises (Brooks, 1986; Stainsby & Brooks, 1990). We, therefore, considered that the utilization of lactate as an energy source for voluntary pedaling exercise lessened the increase in the blood lactate concentration following the superimposing of NMES during low‐intensity voluntary exercises, such as 50% of VT. This unique lactate metabolic circuit induced by the combined application of voluntary exercises and NMES may be useful as physical training for athletes who need to metabolize lactate during athletic events, such as endurance races or intermittent ball games. No significant changes in RPE would be also one of the advantages of the combined application of voluntary exercises and NMES for physical training. The increase in the blood lactate concentration at 125% of VT was significantly greater than those at 50% and 75% of VT (Figure 3). This may indicate that lactate consumption during voluntary exercise is restricted, and the amount of lactate production could exceed that of consumption at 125% of VT. In the present study, even at a high exercise intensity, the increases in the blood lactate concentration following the superimposing of NMES onto the voluntary pedaling exercise were not significantly greater than the net gain in the blood lactate concentration induced by NMES (lower panel in Figure 3). Therefore, an increase in glucose/anaerobic metabolism, which was observed in the present study, could be a simple summation of metabolic responses to voluntary exercise and NMES, and not the result of the augmentation or acceleration of glucose/anaerobic metabolism induced by combining voluntary exercises and NMES. While we could not test the interaction of NMES and exercise intensity in RER because of the limitation of statistical analysis, significant differences in RER between VOL and VOL+NMES were detected at the lowest and highest exercise intensities (lower panel in Figure 2). These results of RER can be interpreted as follows: the glucose/anaerobic metabolism is enhanced regardless of the given exercise intensity.

## 5 PRACTICAL APPLICATIONS

The present study discovered unique metabolic responses during the combined application of voluntary exercises and NMES with changes in given voluntary exercise intensity. As increase in voluntary exercise intensity, increments in oxygen consumption and blood lactate concentration following the superimposing of NMES were decreased and increased, respectively. Also, increments in oxygen consumption following the superimposing of NMES were significantly lower than the net gain in oxygen consumption following NMES when voluntary exercise intensity exceeds the anaerobic thresholds, suggesting that overlapping recruitment of motor units between voluntary exercises and NMES at higher exercise intensity. This unique metabolism may be able to apply to a new type of exercise and we recommend using it at the voluntary exercise intensity under the anaerobic threshold in order to maximize the benefit for additional metabolic responses.

## 6 CONCLUSION

We tested the effect of voluntary exercise intensity on metabolic responses on the combined application of voluntary pedaling exercise and NMES. Our study confirmed that oxygen consumption, the blood lactate concentration, and RER were increased following superimposing NMES onto voluntary pedaling exercise at various exercise intensities. Also, the increments in oxygen consumption following the superimposing of NMES are decreased on exceeding the anaerobic threshold in given voluntary exercises. These results suggest that the metabolic responses during the combination of voluntary exercise and NMES are influenced by the voluntary exercise intensity, and a higher voluntary exercise intensity, such as that exceeding the anaerobic threshold, may be of reduced benefit for additional metabolic responses due to the overlapping recruitment of motor units between voluntary exercises and NMES.

## CONFLICTS OF INTEREST

The authors have no conflicts of interest related to the study.

## AUTHOR CONTRIBUTION

KW: Planned research, conducted experiments, analyzed data, discussed results, and wrote paper. TT and SK: Planned research, discussed results, and edited and reviewed manuscript. TM: Planned research, discussed results, edited and reviewed manuscript.

## References

[phy214758-bib-0001] Bickel, C. S. , Gregory, C. M. , & Dean, J. C. (2011). Motor unit recruitment during neuromuscular electrical stimulation: A critical appraisal. Evaluation studies review. European Journal of Applied Physiology, 111(10), 2399–2407. 10.1007/s00421-011-2128-4. 21870119

[phy214758-bib-0002] Brooks, G. A. (1986). The lactate shuttle during exercise and recovery. Research support, U.S. Gov't, P.H.S. Review. Medicine and Science in Sports and Exercise, 18(3), 360–368. 3523107 10.1249/00005768-198606000-00019

[phy214758-bib-0003] Clamann, H. P. , Gillies, J. D. , Skinner, R. D. , & Henneman, E. (1974). Quantitative measures of output of a motoneuron pool during monosynaptic reflexes. Journal of Neurophysiology, 37(6), 1328–1337. 4436703 10.1152/jn.1974.37.6.1328

[phy214758-bib-0004] Gregory, C. M. , & Bickel, C. S. (2005). Recruitment patterns in human skeletal muscle during electrical stimulation. Physical Therapy, 85(4), 358–364. 15794706

[phy214758-bib-0005] Hamada, T. , Hayashi, T. , Kimura, T. , Nakao, K. , & Moritani, T. (2004). Electrical stimulation of human lower extremities enhances energy consumption, carbohydrate oxidation, and whole body glucose uptake. Journal of Applied Physiology, 96(3), 911–916. 14594864 10.1152/japplphysiol.00664.2003

[phy214758-bib-0006] Hamada, T. , Kimura, T. , & Moritani, T. (2004). Selective fatigue of fast motor units after electrically elicited muscle contractions. Journal of Electromyography & Kinesiology, 14(5), 531–538. 15301772 10.1016/j.jelekin.2004.03.008

[phy214758-bib-0007] Hamada, T. , Sasaki, H. , Hayashi, T. , Moritani, T. , & Nakao, K. (2003). Enhancement of whole body glucose uptake during and after human skeletal muscle low‐frequency electrical stimulation. Journal of Applied Physiology, 94(6), 2107–2112. 12562670 10.1152/japplphysiol.00486.2002

[phy214758-bib-0008] Henneman, E. , Somjen, G. , & Carpenter, D. O. (1965). Functional significance of cell size in spinal motoneurons. Journal of Neurophysiology, 28, 560–580. 14328454 10.1152/jn.1965.28.3.560

[phy214758-bib-0009] Hortobagyi, T. , Lambert, N. J. , Tracy, C. , & Shinebarger, M. (1992). Voluntary and electromyostimulation forces in trained and untrained men. Medicine and Science in Sports and Exercise, 24(6), 702–707. 1602943

[phy214758-bib-0010] Johnson, M. J. , Lortie, G. , Simoneau, J. A. , & Boulay, M. R. (2003). Glycogen depletion of human skeletal muscle fibers in response to high‐frequency electrical stimulation. Canadian Journal of Applied Physiology, 28(3), 424–433. 10.1139/h03-031. 12955869

[phy214758-bib-0011] Jubeau, M. , Gondin, J. , Martin, A. , Sartorio, A. , & Maffiuletti, N. A. (2007). Random motor unit activation by electrostimulation. International Journal of Sports Medicine, 28(11), 901–904. 10.1055/s-2007-965075. 17525881

[phy214758-bib-0012] Kemmler, W. , Bebenek, M. , Engelke, K. , & von Stengel, S. (2014). Impact of whole‐body electromyostimulation on body composition in elderly women at risk for sarcopenia: The Training and ElectroStimulation Trial (TEST‐III). Age (Dordr), 36(1), 395–406. 10.1007/s11357-013-9575-2. 23949160 PMC3889893

[phy214758-bib-0013] Kemmler, W. , Schliffka, R. , Mayhew, J. L. , & von Stengel, S. (2010). Effects of whole‐body electromyostimulation on resting metabolic rate, body composition, and maximum strength in postmenopausal women: The training and electrostimulation trial. Journal of Strength and Conditioning Research, 24(7), 1880–1887. 10.1519/JSC.0b013e3181ddaeee. 20555279

[phy214758-bib-0014] Kemmler, W. , Von Stengel, S. , Schwarz, J. , & Mayhew, J. L. (2012). Effect of whole‐body electromyostimulation on energy expenditure during exercise. Randomized controlled trial. Journal of Strength and Conditioning Research, 26(1), 240–245. 10.1519/JSC.0b013e31821a3a11. 22158139

[phy214758-bib-0015] Kemmler, W. , Weissenfels, A. , Willert, S. et al (2018). Efficacy and safety of low frequency whole‐body electromyostimulation (WB‐EMS) to improve health‐related outcomes in non‐athletic adults. A systematic review. Frontiers in Physiology, 9, 573 10.3389/fphys.2018.00573. 29875684 PMC5974506

[phy214758-bib-0016] Kim, C. K. , Bangsbo, J. , Strange, S. , Karpakka, J. , & Saltin, B. (1995). Metabolic response and muscle glycogen depletion pattern during prolonged electrically induced dynamic exercise in man. Scandinavian Journal of Rehabilitation Medicine, 27(1), 51–58. 7792551

[phy214758-bib-0017] Knaflitz, M. , Merletti, R. , & De Luca, C. J. (1990). Inference of motor unit recruitment order in voluntary and electrically elicited contractions. Journal of Applied Physiology, 68(4), 1657–1667. 2347805 10.1152/jappl.1990.68.4.1657

[phy214758-bib-0018] Koutedakis, Y. , Frischknecht, R. , Vrbova, G. , Sharp, N. C. , & Budgett, R. (1995). Maximal voluntary quadriceps strength patterns in Olympic overtrained athletes. Medicine and Science in Sports and Exercise, 27(4), 566–572. 7791588

[phy214758-bib-0019] Maffiuletti, N. A. , Minetto, M. A. , Farina, D. , & Bottinelli, R. (2011). Electrical stimulation for neuromuscular testing and training: state‐of‐the art and unresolved issues. European Journal of Applied Physiology, 111(10), 2391–2397. 10.1007/s00421-011-2133-7. 21866361

[phy214758-bib-0020] Natsume, T. , Ozaki, H. , Kakigi, R. , Kobayashi, H. , & Naito, H. (2018). Effects of training intensity in electromyostimulation on human skeletal muscle. European Journal of Applied Physiology, 118(7), 1339–1347. 10.1007/s00421-018-3866-3. 29679248

[phy214758-bib-0021] Paillard, T. (2008). Combined application of neuromuscular electrical stimulation and voluntary muscular contractions. Sports Medicine, 38(2), 161–177. 10.2165/00007256-200838020-00005. 18201117

[phy214758-bib-0022] Paillard, T. (2018). Training based on electrical stimulation superimposed onto voluntary contraction would be relevant only as part of submaximal contractions in healthy subjects. Frontiers in Physiology, 9, 1428 10.3389/fphys.2018.01428. 30369886 PMC6194177

[phy214758-bib-0023] Raven, P. , Wasserman, D. , Squires, W. Jr , & Murray, T. (2014). Exercise physiology: An integrated approach. Cengage Learning.

[phy214758-bib-0024] Stainsby, W. N. , & Brooks, G. A. (1990). Control of lactic acid metabolism in contracting muscles and during exercise. Exercise and Sport Sciences Reviews, 18, 29–63. 2162774

[phy214758-bib-0025] Tomczak, M. , & Tomczak, E. (2014). The need to report effect size estimates revisited. An overview of some recommended measures of effect size. Trends in Sport Sciences, 1(21), 19–25.

[phy214758-bib-0026] Vanderthommen, M. , Duteil, S. , Wary, C. et al (2003). A comparison of voluntary and electrically induced contractions by interleaved 1H‐ and 31P‐NMRS in humans. Journal of Applied Physiology, 94(3), 1012–1024. 10.1152/japplphysiol.00887.2001. 12571132

[phy214758-bib-0027] Vincent, W. J. (2005). Statistics in kinesiology. Human Kinetics, 190–192.

[phy214758-bib-0028] Vollestad, N. K. , & Blom, P. C. (1985). Effect of varying exercise intensity on glycogen depletion in human muscle fibres. Acta Physiologica Scandinavica, 125(3), 395–405. 10.1111/j.1748-1716.1985.tb07735.x. 4083044

[phy214758-bib-0029] Wasserman, K. (1984). The anaerobic threshold measurement to evaluate exercise performance. American Review of Respiratory Disease, 129(2 Pt 2), S35–S40. 10.1164/arrd.1984.129.2P2.S35. 6421216

[phy214758-bib-0030] Watanabe, K. , Kawade, S. , & Moritani, T. (2017). Effect of electrode position of low intensity neuromuscular electrical stimulation on the evoked force in the quadriceps femoris muscle. BMC Research Notes, 10(1), 300 10.1186/s13104-017-2630-9. 28728611 PMC5520376

[phy214758-bib-0031] Watanabe, K. , Taniguchi, Y. , & Moritani, T. (2014). Metabolic and cardiovascular responses during voluntary pedaling exercise with electrical muscle stimulation. European Journal of Applied Physiology, 114(9), 1801–1807. 10.1007/s00421-014-2906-x. 24867595

[phy214758-bib-0032] Watanabe, K. , Yoshida, T. , Ishikawa, T. , Kawade, S. , & Moritani, T. (2019). Effect of the Combination of Whole‐Body Neuromuscular Electrical Stimulation and Voluntary Exercise on Metabolic Responses in Human. Frontiers in Physiology, 10, 291 10.3389/fphys.2019.00291. 30949069 PMC6436608

